# The relationships among perceived stress, resilience, sleep quality and first‐month retention of newly employed nurses: A cross‐sectional survey

**DOI:** 10.1002/nop2.1659

**Published:** 2023-02-22

**Authors:** Yueh‐E Lin, Chiu‐Tzu Lin, Mei‐Lien Hu, Sened Tzeng, Li‐Yu Chien

**Affiliations:** ^1^ Department of Nursing Linkou Chang Gung Memorial Hospital Taoyuan Taiwan; ^2^ Department of Nursing Chang Gung University of Science and Technology Taoyuan Taiwan

**Keywords:** 1‐month retention, newly employed nurses, perceived stress, resilience, sleep quality

## Abstract

**Aim:**

Newly employed nurses are subject to high workplace stress, which leads to a low retention rate. Resilience can reduce burnout among nurses. The aim of this study was to explore the relationships among perceived stress, resilience, sleep quality of new nurses during initial employment, and their impacts on first‐month retention.

**Design:**

This is a cross‐sectional study design.

**Methods:**

We used a convenience sampling method to recruit 171 new nurses between January and September 2021. The Perceived Stress Scale, Resilience Scale, and Pittsburgh Sleep Quality Inventory (PSQI) were conducted in the study. Logistic regression analysis was used to explore the impacts on first‐month retention for newly employed nurses.

**Results:**

The initial perceived stress, resilience, and sleep quality of the newly employed nurses were not correlated with the first‐month retention rate. Forty‐four per cent of the newly recruited nurses had sleep disorders. Resilience, sleep quality, and perceived stress of newly employed nurses were significantly correlated. Newly employed nurses assigned to their desired wards had lower perceived stress than their peers.

## 1 INTRODUCTION

Newly employed nurses face many challenges as they transition into practice. It is a stressful time as they have to adapt to complex healthcare practices, a rotating shift work schedule and heavy workload. The reality shock experienced by the new nurses can result in leaving the profession. Therefore, research efforts have been made to the new nurses' perceptions and experiences, and the residency program in order to facilitate a successful transition to the stressful new employment period.

Stress is a well‐recognized phenomenon in the nursing profession, particularly for new nurses. Workplace stress is one of the main reasons nurses resign (Kreedi et al., 2021). Resilience, the ability to remain optimistic in the face of workplace stress, has been proven to be a crucial factor affecting the adaptation of newly employed nurses to the workplace (Kester & Wei, 2018; Meyer & Shatto, 2018) as well as the turnover intention (Cao et al., 2021). New nurse turnover is also associated with poor sleep quality (Han et al., 2020). However, new nurses' stress peaked in the first 2 months (Lin et al., 2020) and their actual turnover rate peaked at the initial stage of employment (Han et al., 2020). These might indicate that more attention needs to be paid to the earlier stage of employment. It remains unknown if the initial stress, resilience and sleep quality of new nurses would affect their initial resign. Understanding the initial stress, resilience, and sleep quality of newly employed nurses, and the impact on their initial retention rate can help nursing managers to plan for new nurse training program and support for newly employed nurses during their initial role transition period.

### 1.1 Perceived stress

Numerous studies have confirmed that newly employed nurses perceive high stress, which may lead to high turnover rates and burnout due to the high workload and lack of professional nursing experience (Chung et al., 2021). Nurses with high levels of perceived stress tend to experience much frustration at work (Sun et al., 2015). Lin et al. (2020) investigated the workplace stress of new employees within the first year of employment; they reported that the stress was the lowest in the first week of employment, and peaked in the first months of employment. Therefore, the stress of newly employed nurses and its influencing factors have always been the issues nurse managers committed to resolve. Many studies have revealed that new nurses are stressed in the first year of employment, but few have examined the initial level of stress with its impact on the first month of employment turnover.

### 1.2 Resilience

Resilience is an individual's ability to remain focused and optimistic about the future in the face of adverse situations (e.g. workplace stress), as well as a critical factor for newly employed nurses to navigate their new careers (Kester & Wei, 2018; Meyer & Shatto, 2018). Resilience has been widely used in various fields for emotional and psychological assessments. Croghan et al. (2021) observed a significant correlation between workplace stress and intention to leave and a negative correlation between perceived stress and resilience of medical personnel. Positive emotions can effectively improve psychological resilience, thereby reducing workplace stress (Kaplan et al., 2017). In addition, highly resilient nurses can actively adapt to stress and adversity (Cooper et al., 2020). Insufficient resiliency among newly licensed nurses has been correlated with the intention to leave (Concilio et al., 2019). Chiang et al. (2021) also reported a significant reduction in resilience level at 3rd month after employment compared with the time of graduation. Resilience is a critical issue facing medical professionals in today's complex medical environment and an essential factor for newly employed nurses to navigate their careers (Kester & Wei, 2018; Meyer & Shatto, 2018).

### 1.3 Sleep quality

First‐line nurses often experience sleep disorders (Tu et al., 2020), and sleep problems are also associated with nursing job performance (Park et al., 2018). Stimpfel et al. (2020) reported that, nurses sleep less than they should, and this may affect their health and clinical performance. Han et al. (2020) investigated the prevalence of sleep disorders in new employees and reported that compared with new nurses without sleep disturbance, those who had sleep disturbance consistently showed a higher turnover in each period within the first 2 years of work. Many studies have revealed that nurses have sleep problems and that sleep quality is related to work performance and retention rates, but few have examined the sleep status of new nurses during the initial stage of employment.

The high workplace stress of newly employed nurses has always been a problem that nurse managers proactively discuss and endeavour to resolve. Although previous studies have reported the correlation between resilience and stress and that between the sleep quality of newly employed nurses and their retention rate, most studies have focused on exploring nurses who are 1–2 years into their careers. By contrast, few studies have explored the factors related to the resilience, stress and sleep quality of newly employed nurses during the introductory period and their correlation with the initial retention rate.

## 2 AIMS

In this study, we investigated the perceived stress, resilience, and sleep quality of newly employed nurses during their initial employment with their first‐month retention rate, and determined the factors influencing their first‐month retention rate. We also provided suggestions regarding training programs and support for new nurses based on the research results.

## 3 METHOD

### 3.1 Research design and sampling

This is a cross‐sectional study design. The study was conducted in a medical centre in northern Taiwan from January to September 2021. Convenience sampling was used to recruit newly employed nurses participating in this study. Data of initial stress, resilience and sleep quality were collected on the first day of orientation before the ward placement, and the retention rate was collected during the first month of employment with an approval from the nursing department of the hospital. We examined the first‐month retention rate because research has shown the new nurse stress level and turnover rate peaked during the first month of employment (Han et al., 2020; Lin et al., 2020), but existing research has rarely investigated the relationships of initial resilience, stress, sleep quality and the first‐month retention rate of new nurses.

G*Power version 3.1.9.4 was used to determine the minimum sample number, we referred to the study results of Chiao et al. (2021) and logistic regression was conducted with a statistical power of 0.8, a significance level (*α*) of 0.05, an odds ratio of 3.316, and a conditional probability (*p*(*Y* = 1)) of 0.878. The minimum sample size was determined to be 58. A total of 173 new nurses were employed in the medical centre during the study period and were all invited to participate. Of these, 171 returned completed questionnaires, resulting in a response rate of 98.8%. SPSS 20.0 was used for all statistical analyses.

### 3.2 Instruments

#### 3.2.1 Demographic questionnaire

The demographic variables of this study included age, religion, nursing degree, reasons for employment (multiple‐choice question), desired placement, prior practice experience in the hospital, and first‐month retention rate.

#### 3.2.2 Perceived stress scale

The Perceived Stress Scale (PSS), developed by Cohen et al. (1983), was used to measure the degree and thoughts of individuals toward stress in life. The original PSS consists of 14 items rated on a 5‐point Likert scale ranging from 0 = “never,” 1 = “rarely,” 2 = “sometimes,” 3 = “very often,” and 4 = “always,” with a total of 0–56 points. The higher the score, the higher the level of perceived stress. The original PSS had a Cronbach's *α* (reflecting internal consistency) of 0.84–0.86. The Chinese version of PSS was translated by Chu and Kao (2005) and has a Cronbach's *α* of 0.85.

#### 3.2.3 Resilience scale for medical workers

Resilience is complex in its concept and theoretical basis, and most questionnaires on resilience have numerous items. This study adopted the Resilience Scale for Medical Workers developed by Hsiao et al. (2019). The scale is intended to measure resilience based on four essential characteristics: meaningful work, acceptance of difficulties, emotional regulation and social relaxation. The Resilience Scale for Medical Workers is a simple self‐assessment tool that individuals can use to assess their own levels of resilience. It comprises 10 items rated on a 5‐point Likert scale, ranging from 1 = “does not apply at all” to 5 = “applies very strongly”, totalling 10–50 points. The higher the score, the higher the resilience. The questionnaire has good reliability (Cronbach's *α* = 0.90) and construct validity (Hsiao et al., 2019).

#### 3.2.4 Pittsburgh sleep quality inventory

The Pittsburgh Sleep Quality Inventory (PSQI) was developed by Buysse et al. (1989), and the Chinese version was translated by Tsai et al. (2005) and used as the tool in this study to measure the overall sleep quality in the past month. The scale comprises 19 items categorized under seven dimensions: subjective sleep quality, sleep latency (i.e. how long it takes to fall asleep), sleep duration, habitual sleep efficiency (i.e. the percentage of time in bed that one is asleep), sleep disturbances, use of sleeping medication, and daytime dysfunction. Each item was scored 0–3 points, totalling a score of 0–21 points. The higher the score, the worse the sleep quality. A total PSQI score of five points was set as the cutoff point. Individuals who received a total PSQI score of ≤5 and >5 were deemed to have good and poor sleep quality, respectively (Buysse et al., 1989). The scale's sensitivity and specificity were 98% and 55%, respectively. Regarding internal consistency, the scale had a Cronbach's *α* of 0.82–0.83 (Tsai et al., 2005).

## 4 RESULTS

### 4.1 Demographic characteristics

Table 1 lists the demographic characteristics of the 171 participants. Their mean age was 22.96 ± 2.61 years. Most nurses were university graduates (91.81%), were assigned to their desired apartments (80.12%), and had internship experience at the hospital (76.02%). Most of them chose to work at the hospital because their classmates, relatives, and friends also worked there (80.12%). The first‐month retention rate was 95.91%.

**TABLE 1:** Demographic characteristics of new nurse participants (*n* = 171).

Characteristic	Mean(SD)	(%)
Age (years)	22.96(2.61)	‐
Religion		
Yes	–	100(58.48)
No	–	71(41.52)
Nursing education		
Junior college graduates	–	14(8.19)
University graduates	–	157(91.81)
Assigned to their desired ward		
Yes	–	137(80.12)
No	–	34(19.88)
Previous internship experience in the hospital		
Yes		130(76.02)
No		41(23.98)
Reason for employment (multiple)		
Relatives or friends also work there		137(80.12)
Close to home		51(29.82)
Recommended by family		35(20.47)
Consider for compensation and benefits		69(40.35)
Job challenges		98(57.31)
The first‐month retention rate		
Yes		164(95.91)
No		7(4.09)

### 4.2 Perceived stress, resilience, and sleep quality

Table 2 presents the scores received by newly employed nurses arriving on the job regarding perceived stress, resilience, and sleep quality. The average score on perceived stress was 1.82 ± 0.48. In terms of resilience, the total score was 39.44 ± 4.98, and the average score was 3.95 ± 0.50. Among the four dimensions of resilience, meaningful work received the highest score of 4.25 ± 0.62. The participants scored 5.60 ± 2.82 on sleep quality, and 43.3% of them had self‐identified sleep disorders (PSQI score > 5 points).

**TABLE 2:** Perceived stress, resilience, sleep quality for newly employed nurses (*n* = 171).

Variables	Mean(SD)	*n* (%)
Perceived stress total score (0–56)	25.43 (6.72)	
Perceived stress average score (0–4)	1.82 (0.48)	
Resilience total score (10–50)	39.44 (4.98)	
Resilience average score (1–5)	3.95 (0.50)	
Meaningful work (1–5)	4.25 (0.62)	
Acceptance of hardness (1–5)	3.91 (0.54)	
Emotional regulation (1–5)	3.75 (0.59)	
Social relaxation (1–5)	3.98 (0.68)	
PSQI		
Time to fall asleep total score (Mins)	29.64 (20.43)	
Sleep duration average score (hours)	7.80 (1.43)	
Global average score (0–21)	5.60 (2.82)	
Sleep disorders average score		
Yes (>5)		74 (43.3)
No (≤5)		97(56.7)

Table 3 presents the results of different demographic groups' perceived stress, resilience, and total sleep scores. University graduates had higher resilience than those who graduated from a junior college (*p* < 0.05); nurses who were assigned to their desired ward had less stress (*p* < 0.001) and lower resilience (*p* < 0.01) compared with those who were not assigned to their desired ward. Regarding reasons for employment, nurses who chose to work in the hospital because it is close to home had a lower mindful awareness score compared with their peers (*p* < 0.05); new nurses who chose to work in the hospital because of compensation and benefits had lower stress (*p* < 0.05) and higher resilience (*p* < 0.05); nurses who chose to work in the hospital because of the job challenges had higher resilience (*p* < 0.05) compared with their peers; nurses without sleep disorders had higher stress (*p* < 0.001), and lower resilience (*p* < 0.01) compared with their peers. No significant differences were noted in perceived stress, resilience, and sleep scores between nurses who stayed and those who resigned within 1 month.

**TABLE 3:** Comparison of demographic profile to the perceived stress, resilience and sleep quality (*n* = 171).

Variables	Perceived stress (0–4)	Resilience (1–5)	Sleep quality (0–21)			
	Mean (SD)	*p*	Mean (SD)	*p*	Mean (SD)	*p*
Religion						
Yes	1.81 (0.50)	0.831	3.95(0.48)	0.849	6.07 (3.06)	0.064
No	1.82 (0.47)		3.94(0.53)		5.26 (2.60)	
Nursing education						
Junior college graduates	1.71 (0.39)	0.386	3.80 (0.21)	0.030*	6.29 (2.64)	0.342
University graduates	1.83 (0.49)		3.96 (0.51)		5.54 (2.84)	
Assigned to their desired ward						
Yes	1.76 (0.47)	<0.001***	5.51 (2.63)	0.008**	3.99 (0.48)	0.399
No	2.06 (0.44)		5.97 (3.54)		3.74 (0.52)	
Previous internship experience in the hospital						
Yes	1.83 (0.50)	0.388	3.93 (0.52)	0.496	5.42 (2.71)	0.153
No	1.76 (0.40)		3.99 (0.43)		6.15 (3.12)	
Relatives or friends also work here						
Yes	1.79 (0.48)	0.151	3.97 (0.48)	0.236	5.64 (2.97)	0.605
No	1.92 (0.47)		3.85 (0.57)		5.41 (2.12)	
Close to home						
Yes	1.85 (0.48)	0.500	3.86 (0.47)	0.137	5.90 (2.61)	0.358
No	1.80 (0.48)		3.98 (0.51)		5.47 (2.91)	
Recommended by family						
Yes	1.84 (0.57)	0.735	4.02 (0.45)	0.312	5.43 (2.67)	0.694
No	1.81 (0.46)		3.92 (0.51)		5.64 (2.87)	
Consider for compensation and benefits						
Yes	1.73 (0.49)	0.042*	4.04 (0.48)	0.042*	5.48 (2.89)	0.654
No	1.88 (0.46)		3.88 (0.51)		5.68 (2.79)	
Job challenges						
Yes	1.76 (0.44)	0.077	4.02 (0.49)	0.018*	5.59 (3.12)	0.979
No	1.89 (0.52)		3.84 (0.49)		5.60 (2.38)	
Sleep disorders						
Yes	2.02 (0.44)	<0.001***	3.83 (0.46)	0.011*	8.18 (2.22)	<0.001***
No	1.66 (0.45)		4.03 (0.51)		3.63 (1.14)	
First‐month retention						
Yes	1.82 (0.49)	0.523	3.95 (0.50)	0.813	5.63 (2.85)	0.400
No	1.76 (0.23)		3.90 (0.49)		4.71 (1.89)	

*p < 0.05; **p < 0.01; ***p < 0.001.

According to the correlation analysis results, resilience, sleep score, and perceived stress of newly employed nurses were significantly correlated. Perceived stress was moderately correlated with resilience (*r* = −0.436, *p* < 0.01). Perceived stress was slightly and positively correlated with sleep quality (*r* = 0.374, *p* < 0.01). Resilience was slightly and negatively correlated with sleep quality (*r* = −0.238, *p* < 0.01; Table 4).

**TABLE 4:** Correlation analysis between the perceived stress, resilience, sleep quality among new nurse (*n* = 171).

Variables	Perceived stress	Resilience	Sleep quality
Perceived stress	–	−0.436**	0.374**
Resilience		–	−0.238**

** *p* < 0.01.

### 4.3 Factors associated with first‐month retention

We conducted a logistic regression analysis to predict initial employment factors influencing newly employed nurses' first‐month retention. The results revealed that entry of personal characteristics (nursing education level), work‐related characteristics (ward placement, organizational compensation and benefits, job challenges), perceived stress, resilience, or sleep quality added a non‐significant amount of variance to the first‐month retention (*χ* ^2^ = 8.746, *p* = 0.271; Table 5).

**TABLE 5:** **Logistic regression analysis of factors related to the first‐month retention** (*n* = 171).

Variables	Beta	SE	Wald	*p*	OR
(Constant)	−17.837	6586.602	0.000	0.998	0.000
Nursing education	−0.771	1.212	0.405	0.524	0.462
Assigned to their desired ward	18.438	6586.600	0.000	0.998	101771479.2
Compensation and benefits	1.494	0.924	2.613	0.106	4.454
Job challenges	0.739	0.911	0.659	0.417	2.095
Perceived stress	0.623	1.116	0.312	0.5276	1.865
Resilience	−1.025	1.079	0.903	0.342	0.359
PSQI	−0.239	0.197	1.461	0.2287	0.3788

*Note*: *χ* ^2^ = 8.746, *p* = 0.271, −2 log likelihood = 49.704, Nagelkerke *R* ^2^ = 0.172.

## 5 DISCUSSION

### 5.1 Resilience

The results revealed that the resilience score of newly employed nurses was 39.44 ± 4.98 (out of 50 points), which was higher than that of other Taiwanese nurses (38.19 ± 5.73; Hsiao et al., 2019). Chiang et al. (2021) also reported a significant change in resilience after 3 months of employment compared with the student stage just prior to graduation (*p* < 0.001). Although the survey in this study was conducted close to graduation time, the resilience questionnaire we used differs from that of Chiang et al. (2021), precluding a direct numerical comparison. Whether the resilience of newly employed nurses changed significantly between the period of arrival and the first 3 months on the job requires further research.

However, the novice stage of nursing is challenging; high resilience helps nurses actively adapt to stress and difficulties (Cooper et al., 2020). Our research results revealed that although the resilience score during initial employment was unrelated to the first‐month retention rate, resilience had a significant negative correlation with stress. It is suggested that resilience can be enhanced by strategies such as promoting initiative, enhancing nursing personnel strengths, fostering personal growth, and cultivating mindfulness practices (Wei et al., 2019). Although it is evident that resilience significantly affected the first‐year turnover intention of new nurses (Cao et al., 2021), the insignificance relationship between resilience score and first‐month retention in this study might indicate that resilience needs more time to show its impact.

### 5.2 Perceived stress

We observed that the newly employed nurses felt pressure “sometimes” or “rarely” during their initial employment. Lin et al. (2020) revealed that during the first 12 months of employment, the employees' stress level was the lowest in the first week of employment and was the highest in the second month of employment, indicating that the stress level of newly employed nurses requires lost attention and monitoring from the initial employment to the second month of employment. Vivian et al. (2019) also indicated that newly employed nurses had higher perceived stress than experienced nurses. Together, these results indicate that the initial employment period is the optimal time for nurses to receive coaching measures related to stress management, considering that their perceived stress has yet to be affected by work factors. Furthermore, in terms of demographic data, newly employed nurses assigned to their desired wards had lower perceived stress than their peers. Existing research has rarely investigated nurses' preferred ward placements when they arrive on the job. Our data indicated that 80.12% of the nurses had the opportunity to be assigned to their desired ward; that is, the nurse manager would consider the preference of newly employed nurses when placing them. This strategy can significantly reduce the perceived stress of nurses arriving on the job, but further investigation is required to confirm its subsequent long‐term effects.

### 5.3 Sleep disturbance

Our data indicated that 43.3% of newly employed nurses arriving on the job had self‐identified sleep disorders (PSQI > 5 points). Park et al. (2018) reported that 79.8% of clinical nurses had sleep disorders. New nurses during initial employment had a lower sleep disorder rate than clinical nurses. The sleep disorder rate of newly employed nurses was significantly higher than that (14.3%) of young Chinese individuals without a medical major of similar age (20.86 ± 1.33; Guo et al., 2016). The results reflect the effect of nursing practice on nurses' sleep (Epstein et al., 2020). Han et al. (2020) reported that compared with new nurses without sleep disturbance, those who had sleep disturbance consistently showed a higher turnover in each period within the first 2 years of work. Although our study result shows no significant difference between the sleep quality of newly employed nurses and the first‐month retention rate, the proportion of newly employed nurses with sleep disorders was still higher than that of their same‐age peers. Therefore, nurse managers could take the time to check on the sleep quality of novice nurses from the start of the new job (Han et al., 2020). Nurse managers may offer healthy work schedules to novice nurses in order to reduce the negative effects of poor sleep. Furthermore, a supportive approach to address sleep problems needs to be implemented during the very early career period, especially the self‐care management strategies for sleep difficulties need to be addressed for newly employed nurses.

### 5.4 The first‐month retention rate

The results revealed that the first‐month retention rate of newly employed nurses was 95.91% (with an actual turnover of 4.19%). Of the studies exploring new nurse turnover, they focused more on turnover intention. For those examining the actual turnover rate, Han et al. (2020) examined the 2‐year trajectories of sleep disturbance and actual turnover among novice nurses. Their study results found that the new nurse turnover rate was 2.6% in the sixth week and peaked at 6.7% from 6 weeks to 6 months. They also found the new nurses who had sleep disturbance consistently had a higher turnover rate than their counterparts. Our study did not find any pre‐employment factors that contribute to the new nurse actual turnover in the first month of employment. Future longitudinal studies might be needed to investigate the long‐term effects of stress, resilience and sleep quality on new nurse retention.

### 5.5 Limitations

The COVID‐19 research results show that nurses were found to experience more stress than other medical staff (Croghan et al., 2021) during the COVID‐19 pandemic, and the pandemic may have exacerbated the new nurses' workplace stress (Crismon et al., 2021; García‐Martín et al., 2021). This study was conducted during the COVID‐19 pandemic, our results may only reflect the condition of newly employed nurses during the COVID‐19 pandemic. This was a cross‐sectional study conducted in a medical centre using convenience sampling, which limits the inference drawn from the results. Future longitudinal studies are required to elucidate the changing trends in the resilience and sleep quality of new employees. Furthermore, the first‐month retention rate reported in this study is high, the celling effect may occur to make it difficult to reflect an accurate measure of the results.

## 6 CONCLUSION

Perceived stress, resilience, and sleep quality scores of new nurses during the initial employment period were found to be significantly correlated, but the scores of these three variables were not correlated with the first‐month retention rate. Newly employed nurses assigned to their desired wards had lower perceived stress compared with their peers. The proportion of newly employed nurses with sleep disorders was higher than that of their peers.

## 7 IMPLICATIONS FOR NURSING

Medical personnel are constantly working in a high‐stress environment, which increases burnout, which may entail negative emotions and affect workplace safety. The resilience and perceived stress of newly employed nurses during the introductory period have not been affected by work factors, which suggests that the introductory period is the optimal time for implementing interventions to maintain or improve resilience. Nurse managers can first monitor the resilience and stress status of new nurses and conduct an early intervention to assist new nurses in successful role transitions. A comprehensive strategy might involve stress management, mindfulness, and resilience training (Chesak et al., 2019; Concilio et al., 2021; Contreras Sollazzo & Esposito, 2020; Stacey et al., 2020). Nurse resilience can also be improved through formal education, social support and meaningful recognition at work (Kester & Wei, 2018).

We observed that 43.3% of newly employed nurses had self‐identified sleep disorders during the introductory period following initial employment. Because sleep disorder considerably affects nurses' burnout and care quality, nurse managers are advised to conduct monitoring of newly employed nurses' sleep for early diagnosis of sleep disorder. Flexible work arrangements should be made for nurses who have sleep disorders to reduce the effects of multishift scheduling and care stress on sleep. In addition, nurse managers should assist all newly employed nurses in learning about self‐care management measures that improve sleep quality as soon as possible (Han et al., 2020). Self‐care guidance helps newly employed nurses to improve sleep quality and thereby reduce the high incidence of sleep disorders among nurses in the future.

## AUTHOR CONTRIBUTIONS

YEL, MLH, ST and LYC have made substantial contributions to conception and design, or acquisition of data, or analysis and interpretation of data. YEL and LYC have been involved in drafting the manuscript or revising it critically for important intellectual content. YEL, CTL, HLH, ST and LYC have given final approval of the version to be published. Each author should have participated sufficiently in the work to take public responsibility for appropriate portions of the content. YEL, CTL, HLH, ST and LYC Agreed to be accountable for all aspects of the work in ensuring that questions related to the accuracy or integrity of any part of the work are appropriately investigated and resolved.

## FUNDING INFORMATION

This work was supported by the Chang Gung Medical Foundation at Linkou Branch [grant numbers CMRPG3K1671, 2021].

## CONFLICT OF INTEREST STATEMENT

None of all authors.

## RESEARCH ETHICS COMMITTEE APPROVAL

This study was approved by the Institutional Review Board of Chang Gung Memorial Hospital (case number 201902131B0C501). The researchers explained the study objectives and procedures to the participants and only proceeded with the research after obtaining their written informed consent. The research participants had the right to withdraw at any time without compromising work or personal rights. All questionnaires were anonymously coded, and any data were preserved to ensure the anonymity and privacy of participants.

## Data Availability

The data that support the findings of this study are available from the corresponding author upon reasonable request.
